# The influence of teacher leadership on students academic burnout in China: potential multiple mediating effects of meeting basic psychological needs

**DOI:** 10.3389/fpsyg.2025.1558159

**Published:** 2025-08-27

**Authors:** Anqi Fang

**Affiliations:** ^1^School of Educational, Huazhong University of Science and Technology, Wuhan, Hubei, China; ^2^Mandarin Training and Testing Station, Changsha Education College, Changsha, Hunan, China

**Keywords:** teacher leadership, academic burnout, basic psychological needs, self-determined motivation, multiple mediating effects

## Abstract

Teacher leadership has an important influence on students' academic burnout. Based on the theory of self-determination, this paper constructs an analytical framework of “external environmental resources-internal psychological needs satisfaction” to reduce students' academic burnout. Taking senior high school students in China as samples, it explores the potential effect and mechanism of China teachers' leadership on students–academic burnout. The results show that teacher leadership can not only directly and negatively affect students' academic burnout, but also further negatively affect academic burnout through the parallel mediation of learning autonomy, self-efficacy and teacher-student relationship. Also, the teachers' teaching leadership is an important factor affecting students' academic burnout, and the three psychological needs - autonomy, competence and relationship—have independent functional values on students' academic burnout. Therefore, constructing a good teacher's teaching leadership system can meet students' psychological needs of autonomy, competence and relationship, thus helping to reduce students' academic burnout.

## 1 Introduction

When students lack interest or motivation in learning but are forced to do so, they will feel bored, tired, depressed and frustrated, leading to a series of inappropriate behaviors of avoiding learning. This state is called learning burnout (Freudenberger, 1974; Pines et al., 1981; Yang, 2004). This state is manifested as a persistent, negative, and learning-related psychological state among high school students, which is specifically manifested in the decline of learning motivation, alienation from learning activities, and a lack of sense of achievement and enjoyment in learning (Zhang and Zhao, 2018). Existing research has revealed that academic burnout will not only lead to the decline of academic performance, but also have a profound negative impact on students' mental health. Once students fall into academic burnout, they may suffer from psychological problems such as depression and anxiety, and may be accompanied by a series of bad behaviors. Therefore, it is of great practical significance to explore influence factors and internal mechanisms causing academic burnout among high school students.

Students' engagement and academic burnout are usually considered as the opposite poles of the same continuum. In this view, burnout is simply a lack of emotional, behavioral, and cognitive engagement. Generally speaking, students' engagement and burnout are “everything to everyone”. The phenomenon of students' academic burnout is the result of multiple factors, including their internal actors, such as motivation, self-efficacy, personality traits and needs (Luo et al., 2014; Ling et al., 2014), as well as external environmental factors including school cultural atmosphere and social support from family, teachers and peers. The research has shown that teachers can alleviate students' academic burnout by implementing important strategies by promoting their academic self-confidence and self-efficacy (Patrick et al., 2011; Federici and Skaalvik, 2014).

There are some empirical research to support the relationship between teacher leadership and student engagement. For example, a group of scholars have assured that schools with special emphasis on joint leadership efforts encourage community members to have a common goal in learning, thus improving students' learning outcomes (Flores et al., 2013). A common fact revealed in the research showed that the teacher with leadership usually promote and practice teaching method centering on students interests and needs. Teachers with leadership focus on meeting students' needs by using attractive and innovative teaching methods and strategies, rather than relying solely on textbook coverage (Donnelly et al., 2019; Hovardas, 2016; Wenner and Campbell, 2017). Scholars have also mentioned the focus on students' well-being and learning (Huang, 2016), should be at the center of all school teachers. Teachers with leadership are motivated to enrich their professional knowledge. In order to meet the needs of students, teacher with leadership can take different actions, such as differentiated teaching (Donnelly et al., 2019), and extracurricular tutoring (Gordon and Solis, 2018; Yu, 2015). Teaching leadership is a key ability of teachers and is of great value to the comprehensive development of students (Huang and Wu, 2020). By strengthening leadership, teachers pay more attention to students' subjectivity, and carry out teaching based on students' thinking and behavior which has a positive impact on students' learning motivation, curiosity and self-confidence. From the perspective of the relationship between teacher leadership and students' burnout, some scholars believe that teacher leadership is positively associated with academic resilience and motivation. Academic resilience negatively predicts burnout (Trigueros et al., 2020).

Overall, there are currently limited researches on the mechanism of the influence of teachers teaching leadership on students' academic burnout. In order to deeply understand the internal mechanism of how teachers' support affects academic performance, some scholars have conducted empirical studies on the relationship between teachers' support, satisfaction of basic psychological needs, internal motivation, and academic interaction. However, there is a lack of research on the independent mediating and chain mediating role of the satisfaction of psychological needs and the internal motivation on influencing the relationship between teacher leadership and students' academic performance, and there are few studies exploring the mechanism of teacher's teaching leadership influence on students' academic burnout.

The study on student's academic burnout instead of academic achievements is of research value and practical significance. Previous studies have proved that students' perception of teachers' support will affect their autonomous motivation because their basic psychological needs are satisfied, which further affects their learning burnout (Luo et al., 2014). According to the theory of self-determination, the nourishment and support from external environment are crucial to transform external motivation into internal motivation. This transformation process occurs when the external environment meets the individual's needs for competence, autonomy and relationship (Ryan and Deci, 2020), and teachers' leadership behavior, as a supporting factor from external environment, can be transformed into internal motivation to help stimulate students. Therefore, this study aims to explore the potential influence of high school teachers' teaching leadership on students' academic burnout, and use self-determination theory to analyze how academic efficacy, teacher-student relationship, and learning autonomy play a role in the process of teachers' teaching leadership affecting students' academic burnout. By gaining a deeper understanding of these mechanisms, effective strategies can be sought to alleviate students' academic burden, reduce the incidence of academic burnout, and thereby enhance students' learning motivation.

## 2 Literature review and research hypothese

### 2.1 Self-determination theory

Self-Determination Theory (SDT) is a theory about human motivation proposed by Edward L. Deci and Richard M. Ryan in the 1980s. The theory posits that human behavior is primarily motivated by three fundamental psychological needs: autonomy, competence, and relatedness. All three needs must be satisfied simultaneously to promote intrinsic motivation.

Based on the review of the existing literature, Chen et al. (2020) have made in-depth research on the theory of basic psychological needs focusing on autonomy, ability and relevance, and discussing them as independent variables or dimensions. It is pointed out that the research of the satisfaction of one-dimensional basic psychological needs mainly focuses on three perspectives: independence, coordination and balance. Ryan and Deci (2000) emphasized that there are dynamic interactions among the three basic psychological needs, and each basic need has its own independent functional value. At present, the dynamic educational function and independent functional value of the three basic psychological needs have been supported by numerous empirical studies. For example, the researches reported by Cerasoli et al. (2016) and Dysvik et al. (2013), etc.

### 2.2 The impact on students' academic burnout

In previous studies, the exploration of teaching leadership often focused on principals and other teaching administrators. However, as the research perspective shifts from administrative management to classroom teaching (Patrizio and Stone-Johnson, 2016), teachers' teaching leadership began to receive widespread attention from researchers. Teachers' teaching leadership includes a series of positive interpersonal skills, which can stimulate students' enthusiasm for learning, provide positive guidance, point out the correct learning direction, and help students overcome obstacles during learning process (Ryan and Deci, 2020). This kind of leadership plays a key role in promoting students' learning, growth, and development. Self-determination theory puts forward that individual behavior is driven by three core internal psychological needs, namely autonomy, competence and relationship needs. These needs are crucial for stimulating and maintaining internal motivation, promoting the integration of external motivation and internal motivation, cultivating individual's internal desire, and satisfying emotional needs (Chen, 2005). In the research of teachers' teaching leadership, the support provided by teachers, such as stimulating students' interest in learning, promoting high-quality educational behavior, providing positive guidance and pointing out the correct learning direction, helping students overcome learning obstacles, and encouraging students to enhance their learning satisfaction, are regarded as the key factors to meet students' basic psychological needs. This kind of support helps to transform students' external motivation for engaging in learning activities into internal motivation, thus effectively reducing academic burnout. The research shows that teachers' different leadership styles can create a favorable environment for teaching, and include a variety of teaching strategies to promote students' continuous engagement in learning activities (Wang, 1995; Zhang, 1996; Fleishman and Harris, 1962). Baker III theory focuses on how teachers can reduce academic burnout by influencing students' cognition of learning goals, personal goals, and ways to achieve them (Baker, 1996).

### 2.3 The mediating role of learning autonomy, academic efficacy, and teacher-student relationship

According to the theory of self-determination, an individual's intensity of needs is the result of their interaction with social environment, and this intensity of needs can be used to directly or indirectly predict an individual's personality characteristics, behavior, or psychological state (Ryan and Deci, 2000). This view reveals that the influence of social environmental factors on individual's internal characteristics, behavior, and psychological state does not occur directly, but may have subsequent effects by meeting individual's basic psychological needs. Ryan and Deci (2008) research found that when the social environment effectively meets the basic psychological needs of individuals, people tend to internalize and integrate various aspects of the social world. However, when the social environment hinders the satisfaction of these needs, the internalization efficiency of individuals will decrease. That is to say, when social environment allows individuals to develop their own abilities, establish interpersonal relationships, and act in a self-approved way, they are more likely to integrate the structure transmitted by society into their self-concept. Based on this theoretical perspective, this study speculates that, as an environmental support variable, its influence on academic burnout may also be achieved by meeting the basic psychological needs of individuals. In other words, the influence of teachers' teaching leadership on students' academic burnout may be achieved through the mediating role of satisfying basic psychological needs.

Learning autonomy refers to learners' ability to take responsibility for their own learning process. Learners with autonomous learning ability can independently set learning goals, contents, materials, and methods, independently arrange their own learning time, location, and progress, and self-evaluate learning outcomes (Holec, 1981). Littlewood's research points out that this kind of ability includes not only learners' ability to control their own learning strategies and skills, but also learners' subjective desire and self-confidence in controlling their own learning process (Littlewood, 1996). In the field of education, students' autonomy is very important (Ryan and Niemiec, 2009). Research on the theory of learning autonomy has shown that the degree of autonomy achievement is restricted by the ability of both students and teachers (La Ganza, 2008; Little, 2007; Schwienhorst, 2009). In order to effectively cultivate students' autonomy, teachers need to explore in depth how to use various favorable factors in teaching practice to provide learners with the support conditions needed for autonomous learning; How to guide learners to adopt scientific and effective learning strategies, and cultivate positive learning motivation and self-esteem (Du, 2019).

Numerous empirical studies have shown the importance of teachers' teaching support in promoting learner autonomy. For example, Xu and Peng (2004) and Lin (2008) found through their investigation that college students' autonomous learning ability is generally insufficient, and they expect teachers' intervention and guidance. In addition, the research results of educational projects aimed at improving learners' autonomy also reveal the key role of teachers in the process, indicating that teachers' contribution to promoting learners' autonomy cannot be ignored (He, 2005; Li and Zhang, 2006; Ouyang and Xie, 2009; Lin and Chen, 2009; Liu, 2009). According to the theory of self-determination, when individuals' autonomous needs are met, they tend to generate more internal motives, thus showing positive behaviors and attitudes (Ryan and Deci, 2000). In the field of learning, this theory has been supported by empirical evidence: individuals with lower learning burnout often have higher autonomous learning motivation (Salmela-Aro and Upadyaya, 2014). Individuals with clear learning goals often show a lower level of learning burnout, while those who avoid academic achievements show a higher level of learning burnout (Shi et al., 2012). Youkun Wang's study on 481 non-English major college students shows that students with stronger autonomous learning ability often experience a lower level of learning burnout (Wang and Wang, 2019). In addition, the study conducted by Zhao et al. (2014) on nursing undergraduates also reveals a significant negative correlation between learning burnout and autonomous learning ability. These findings emphasize the potential value of autonomous learning ability in alleviating learning burnout.

Bandura (1997) pointed out that efficacy refers to an individual's belief in their ability to organize and execute actions necessary to produce certain achievements or outcomes. This is an individual's subjective judgment of his/her success in completing an activity, that is, his/her confidence in his/her ability to achieve behavioral goals in a specific field Bandura, (1993). In the academic field, this belief is embodied as academic self-efficacy, which includes two dimensions: self-efficacy in learning ability and self-efficacy in learning behavior. Self-efficacy in learning ability involves an individual's evaluation of his/her ability to complete his/her studies, achieve excellent grades, and avoid academic failure. The self-efficacy in learning behavior focuses on students' evaluation of whether their learning behavior can achieve their learning goals, that is, their expectations of the results of their behavior (Wang and Xin, 1999). As an important internal motivation variable and internal resource, self-efficacy significantly affects an individual's behavior (Bandura and Walters, 1977).

Teaching leadership is a comprehensive ability displayed by teachers in the daily teaching practice in schools, which involves all those abilities that directly or indirectly promote teaching and influence students' learning (Zhang and Zhao, 2018). Research has shown that teachers with transformational leadership can enhance students' self-efficacy by encouraging and motivating their verbal behaviors, and at the same time help students achieve success and increase their successful experience through intellectual stimulation. These leadership behaviors all contribute to improving students' self-efficacy. In addition, students who experience the charm of teachers' transformational leadership will regard teachers as role models for leadership development, and teachers' successful leadership as an alternative experience can also enhance students' self-efficacy. Multiple empirical studies have confirmed that the four behavior patterns of transformational leadership can effectively and positively predict subordinates' self-efficacy (Sui et al., 2012; Meng et al., 2011). In the research of China scholars, they used a nationwide student achievement evaluation data to explore the relationship between teachers' teaching leadership and school achievement, and added the students' sense of efficacy in learning as a consideration factor in the student achievement part (Zheng et al., 2017).

The academic community widely recognizes efficacy as a key driving factor for individuals to perform specific behaviors. Numerous empirical studies have also confirmed that the individual's sense of efficacy has an important influence on his/her behavior and reaction in study, work and emotional regulation (Zhou and Guo, 2006). For example, Cuirong Wang's research reveals a significant negative correlation between learning self-efficacy and academic burnout among vocational college students (Wang, 2008). In addition, researchers such as Zhu and Wang (2009), Wang and Miao (2012), and Chen and Yang (2018)) also found that students' self-efficacy can effectively predict their academic burnout, showing a negative predictive effect. This shows that self-efficacy is not only an important factor to stimulate the individual's internal motivation, but also profoundly affects the individual's participation and engagement level in various activities (Gao, 1998). The study further points out that by improving students' learning efficacy, it can play a positive role in strengthening learning motivation, adjusting learning strategies, and improving academic performance, and at the same time, it can also effectively alleviate students' learning burnout to a certain extent (Zhou, 2016).

Teacher-student interaction has always been a hot topic in and outside the field of education, with a long history of importance. Krupskaya, a famous educator in the Soviet Union, once profoundly pointed out:

“The core of education lies in building the actual relationship and system between individuals and collectives and society to ensure that individuals can be socialized smoothly” (Shao, 2008).

Classroom is a complex social system, in which the relationship between teachers and students is also a complex structure (Pianta et al., 2012). Lan and Moscardino (2019) define the connotation of teacher-student relationship as a comprehensive embodiment of intimacy and conflict level between teachers and students. This relationship is not only a link between teachers and students, but also plays a decisive role in the process of students' school adaptation. Lots of research literature has emphasized the influence of teacher-student relationship on individual adaptation.

In the educational environment, teacher leadership is a key factor in shaping a good teacher-student relationship (Wang, 2019). Research has shown that transformational leadership belongs to “moral leadership” and “relationship leadership” (Zhang, 2008), among them, “relationship leadership” emphasizes the symbiotic interaction between leaders and followers, which is a mutually reinforcing collective behavior. Transformational leadership, as an external environmental factor, has a positive impact on the improvement of teacher-student relationship (Tian et al., 2021). When the relationship between teachers and students is close, teachers can carry out teaching activities more effectively. Therefore, close teacher-student relationship can enable teachers to provide more effective teaching (Nel, 1992).

The teacher-student relationship is widely regarded as a key factor of students' emotional security and learning motivation (Bowlby, 1980; Howes et al., 1994; Pianta, 1999). Empirical research has revealed that the teacher-student relationship, as an external environmental factor, has an impact on students' academic burnout through a series of internal mediating mechanisms (Yavuz and Kutlu, 2016). Specifically, a positive teacher-student relationship can reduce the incidence of academic burnout by stimulating positive emotional responses from students toward teachers (Chen and Wu, 2022; Ljubin-Golub et al., 2020; Hatzenbuehler, 2009). There is a significant correlation between teacher-student conflicts and high levels of academic burnout, as well as low levels of subjective wellbeing (Yu, 2015). When teachers provide more support in teaching and create an atmosphere of self-supporting teacher-student relationship, students tend to show higher academic engagement and subjective well-being. Research has shown that the higher level of teachers' support perceived by students, the more harmonious the teacher-student relationship would be, the more active the students would be engaged in learning, and correspondingly, the lower the level of academic burnout (Roorda et al., 2011; Marion et al., 2014). It is worth noting that the role of teacher-student relationship in predicting academic burnout even exceeds that of peer relationship (Zhang and Gao, 2019; Wang et al., 2023). Therefore, it can be concluded that a good teacher-student relationship can not only enhance students' willingness to learn, but also significantly reduce the risk of academic burnout.

### 2.4 Integration of theory

In this study, we deeply analyzed the root causes of students' academic burnout, and put forward a new point of view: academic burnout is the result of the combined effects of external environmental factors and individual factors. This study holds that when the external environment can meet the three major psychological needs of individual autonomy, competence and relationship, students' learning enthusiasm will be improved, and academic burnout will be reduced accordingly. Specifically, learning autonomy refers to the individuals' clear understanding of the will and consistency of their own behaviors, reflecting the individuals' sense of ownership over their own behaviors. Competency needs refer to the ability of individuals to feel effectiveness in their actions, that is, when facing environmental challenges, they can effectively cope with and achieve the expected goals; Relationship needs to refer to the degree of closeness between individuals and others. The satisfaction degree of basic psychological needs is defined as the satisfaction degree of external environment to individual basic psychological needs (Ryan and Deci, 2017). By expounding the concepts of learning autonomy, learning efficacy, and teacher-student relationship, we can find that in the context of education and teaching, these three aspects can reflect the satisfaction degree of the three major psychological needs of autonomy, competence and relationship to a certain extent.

**Figure 1:**
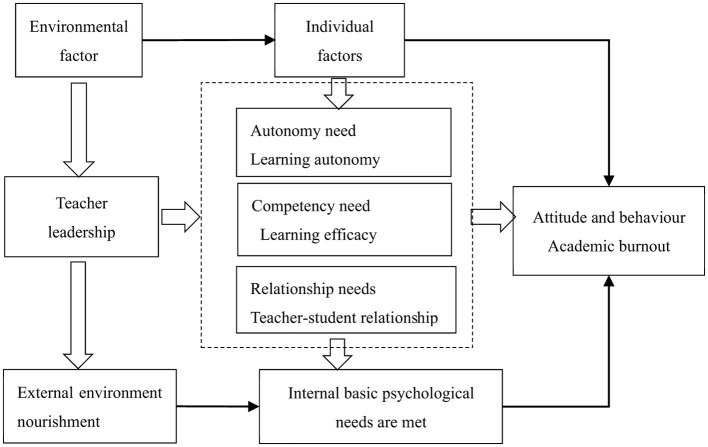
Analytical framework of exploring the associations between teacher leadership and students' academic burnout in China.

Some studies have conducted comprehensive research on basic psychological needs, while others have studied them as independent variables or dimensions (Chen et al., 2020). Ryan and Deci (2000) pointed out that we should not only recognize the independent functional value of each basic need, but also not ignore the dynamic interaction between them. Based on this point of view, this study constructs a theoretical analysis framework as shown in Figure 1 to explore how teachers' teaching leadership can meet students' three psychological needs of autonomy, competence, and relationship to improve students' internal motivation for learning and reduce academic burnout.

In summary, this study proposes the following hypothesis:

H1: The leadership of high school teachers in China has a negative impact on students' academic burnout.
H2: Learning autonomy plays a mediating role in the relationship between teachers' leadership and students' academic burnout in high schools in China.
H3: Self-efficacy plays a mediating role in the relationship between teachers' leadership and students' academic burnout in high schools in China.
H4: Teacher-student relationship plays a mediating role in the relationship between teachers' leadership and students' academic burnout in high schools in China.

## 3 Methods

### 3.1 Samples

Four senior high schools (including two key high schools and two ordinary high schools) from a southern province of China were selected by a convenient sampling method. These four schools are located in a province that is currently in the exploratory and experimental period of the new college entrance examination reform, and are actively implementing reform measures to improve students' learning attitude and behavior. Two classes were randomly selected from each school to distribute questionnaires for investigation and research. The data were collected through a paper-based questionnaire survey conducted in classroom settings during regular school hours. Trained researchers administered the questionnaires, first explaining the study's purpose, voluntary nature, and confidentiality to participants. Students were assured that their responses would remain anonymous, with no identifying information collected, to encourage honest and unbiased answers. Informed consent was obtained from school administrators and participating students prior to data collection, adhering to ethical research standards. The survey took approximately 15-20 minutes to complete, and researchers were present to address any questions, ensuring clarity and consistency in the process. A total of 466 questionnaires were distributed, and 435 valid questionnaires were collected, with an effective rate of 93.35%, including 203 boys and 232 girls.

Given the lack of accurate estimates of the overall effect size in the early stages of this study, we referenced existing literature on the relationship between information technology use and innovative behavior to support the reasonable determination of sample size. Huang et al. (2025) noted in “The Contagious Effect of Perceived Teacher's Job Burnout on High School Student's Study Burnout: A Resource-loss Perspective” that there is a significant positive correlation between high school students' perception of teachers' negative work states and academic burnout (*r* = 0.11, *p* < 0.01). Given that this study shares high similarity with our research in terms of research topic, measurement methods, and sample characteristics, it holds strong reference value. We therefore conducted a G*Power power analysis based on this study. Due to the lack of precise estimates for overall parameters in the early stages of our research, and given the similarities between the research methods and sample characteristics of this literature and our study, we also conducted a G*Power power analysis based on this study. According to the results of G*Power 3.1, under the conditions of effect size *r* = 0.11, significance level α = 0.01 (two-tailed test), and actual sample size *N* = 435, the statistical power (Power, 1−β) of this study reached 0.892, significantly higher than the commonly used 0.80 standard in social science research. This indicates that the current sample size has a high level of sensitivity and can reliably test the weak effects in the research hypothesis. However, the determination of the current sample size primarily relies on reference to effect sizes in the literature, which still has certain limitations. Future research may consider conducting small-scale preliminary pre-experiments to obtain actual data, thereby more accurately estimating the overall effect size, and combining statistical tools such as G*Power for sample size prediction analysis to further enhance the scientific rigor of the research design and the stability of the results.

### 3.2 Measurement tools

The content of the complete questionnaire used in this study can be found in Appendix A of the Supplementary material. The questionnaire's question set basically followed the following principles.

The “Academic Self-efficacy Scale” compiled by Yusong Liang and Zongkui Zhou in 2000 is adopted for learning efficacy, which refers to the relevant dimensions in the academic self-efficacy questionnaire compiled by Pintrich and De Groot (1990). The scale contains 22 questions, which are divided into two dimensions: self-efficacy of learning ability and self-efficacy of learning behavior. The scale adopts a five-point scoring system. The Cronbach's alpha coefficient of the total scale is 0.89, and the Cronbach's alpha coefficient of the two dimensions are 0.820 and 0.752, respectively.

The measurement of academic burnout is based on Maslach and Jackson's (1986) three-dimensional model. Wu et al. (2010) took Chinese adolescents as research objects and developed the “Adolescent Academic Burnout Scale”, which covers three aspects: physical and mental fatigue, academic alienation, and low sense of achievement. There are total of 16 items, of which 4 belong to emotional exhaustion dimension, 5 belong to academic alienation dimension, and 7 belong to low sense of achievement dimension. Using the five-point scoring method, the Cronbach's alpha coefficients of the three sub-scales in the study are 0.77, 0.80, and 0.86, respectively, and the Cronbach's alpha coefficient of the total scale is 0.86.

The “Teacher's Teaching Leadership Scale” was developed by Baker (1996) and compiled by Yijun Chen. The scale contains 33 questions, of which 4 belong to the dimension of arousing students' desire for learning, 9 belong to the dimension of achieving quality educational behavior, 6 belong to the dimension of providing positive guidance and correct learning direction, 8 belong to the dimension of helping students eliminate learning obstacles, and 5 belong to the dimension of encouraging students to increase their learning satisfaction. The scale adopts a five-point scoring system. The Cronbach's alpha coefficient of the total scale is 0.9553, and the Cronbach's alpha coefficients of the five dimensions are 0.7738, 0.8529, 0.8161, 0.8752, and 0.8616, respectively.

The teacher-student relationship scale adopts the “School Atmosphere Questionnaire” compiled by Yibing Yu and Minggui Ge. The scale takes the school environment classification theory (Moos) as the theoretical basis, and consists of 38 questions in five dimensions: teacher-student relationship (9 questions), classmate relationship (7 questions), order and discipline (7 questions), academic pressure (8 questions), and development diversity (7 questions). These questions use a four-point scoring method, and the Cronbach's alpha coefficient of the questionnaire is 0.857. Considering the research purpose, this study adopts the subscale of teacher-student relationship in the “School Atmosphere Questionnaire”, and the Cronbach's alpha coefficient of the dimensions of teacher-student relationship is 0.91. As a dimension analysis, it verifies the scientific validity of the teacher-student relationship scale, mainly from the aspects of students' trust, respect, equality, fairness, expectation and intimacy to perceive whether teachers trust and respect students, treat students equally, and have appropriate expectations, and whether students are willing to treat teachers as friends.

The “Autonomous Learning Questionnaire for High School Students” was compiled by Weiguo Pang, which is currently the most widely used scale for measuring autonomous learning ability in China, which measures students' autonomous learning level. The questionnaire uses Likert 5-point scoring method to evaluate students' autonomous learning from aspects such as learning motivation, learning content, and learning environment. Cronbach's alpha coefficient is 0.96.

### 3.3 Research methods

This study employed SPSS 26.0 for statistical analysis and utilized Mplus 8.0 to construct structural equation models (SEM) for assessing convergent validity and discriminant validity, modifying model fit indices, and testing research hypotheses. During the SEM development, relationships among variables were thoroughly investigated, with specific focus on analyzing the mediating effects of learning autonomy, academic self-efficacy, and teacher-student relationships. The mediation analysis followed the testing procedures established by Fang et al. (2014) and Wen and Ye (2014). The modeling sequence proceeded as follows: First, Model 1 examined the direct effect of teacher leadership on student academic burden, with teacher leadership as the independent variable and academic burden as the dependent variable; Second, Models 2, 3, and 4 respectively tested the independent mediating roles of learning autonomy, academic self-efficacy, and teacher-student relationships in the relationship between teacher leadership and academic burden, with each mediator analyzed in a separate model.

## 4 Research results

### 4.1 Common variance test

The common method deviation of data is tested by Harman single factor model method. In this test, the un-rotated principal component analysis is carried out on all the variables simultaneously. The results show that there are 12 factors with characteristic values greater than 1, and the first factor extracted explains 19.41% of the total variation, which is below the critical value of 40%, indicating that there is no significant common method deviation.

### 4.2 Data processing

This study utilized Mplus 8.0 software to assess the overall fit of the structural model. SPSS 26.0 and its PROCESS macro program were employed to examine the direct effect of teachers' instructional leadership on high school students' academic burnout, as well as the mediating effects of learning autonomy, academic self-efficacy, and teacher-student relationships. Prior to conducting formal statistical analyses, the study performed necessary preliminary assumption tests on the data to ensure the validity and reliability of the analysis results. Specifically, the study plotted scatter plots to observe trends among variables and preliminarily assess whether the data met the assumption of linear relationships. Additionally, the variance inflation factor (VIF) was calculated for each independent variable to detect potential multicollinearity issues. The results indicated that the VIF values for all independent variables were far below 10 (the VIF values for each variable were: teachers' instructional leadership [2.187], learning autonomy [2.041], academic self-efficacy [2.338], and teacher-student relationship [1.023]), indicating that there were no serious multicollinearity issues. Besides, regression assumptions, including independence (Durbin-Watson statistic), homoscedasticity (Breusch-Pagan test), normality, linearity, and multicollinearity (VIFs), were tested and met, ensuring the validity of the mediation analyses.

### 4.3 Descriptive statistics and correlation analysis

**Figure 2:**
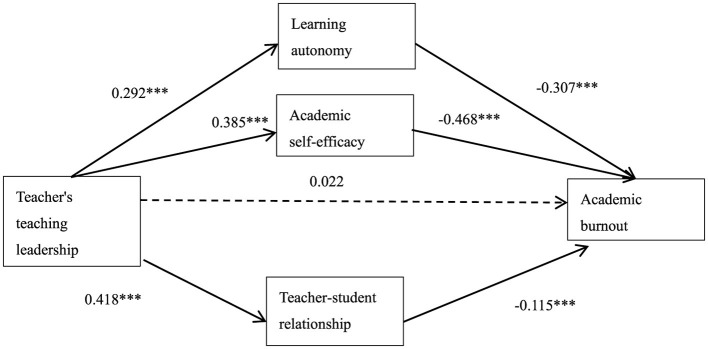
The mediating model of learning autonomy, learning self-efficacy, and teacher-student relationship (for specific path coefficients and effect sizes, see Table 3). ^***^p < 0.001.

The descriptive statistics and correlation analysis results among the variables are shown in Table 1. According to Table 1, teachers' teaching leadership is positively correlated with learning autonomy (*r* = 0.377, *p* < 0.001), academic self-efficacy (*r* = 0.517, *p* < 0.001) and teacher-student relationship (*r* = 0.614, *p* < 0.001). Teachers' teaching leadership (*r* = −0.389, *p* < 0.001), learning autonomy (*r* = −0.644, *p* < 0.001), academic self-efficacy (*r* = −0.697, *p* < 0.001), and teacher-student relationship (*r* = −0.320, *p* < 0) are all significantly negatively correlated with academic burnout. The significant correlation between variables satisfies the premise of mediation test. These correlations align with the theoretical framework depicted in Figure 2, supporting the hypothesized relationships among teacher leadership, learning autonomy, academic self-efficacy, teacher-student relationship, and academic burnout, as outlined in Hypotheses H1-H4 (Section 2.4).

**Table 1:** Descriptive statistics and correlation analysis among variables (*n* = 435).

**Variables**	**Academic burnout**	**Teacher's teaching leadership**	**Learning autonomy**	**Academic self-efficacy**	**Teacher-student relationship**
Academic burnout	One			
Teacher's teaching leadership	-0.389***	One		
Learning autonomy	-0.644***	0.377***	One	
Academic self-efficacy	-0.697***	0.517***	0.670***	One	
Teacher-student relationship	-0.320***	0.614***	0.354***	0.250***	One
Average value	2.913	3.684	3.186	3.501	2.797
Standard deviation	0.648	0.771	0.626	0.669	0.524

^***^p < 0.001.

Skewness and kurtosis for all variables were assessed, with values within acceptable ranges (skewness < |2|, kurtosis < |7|), supporting approximate normality.

### 4.4 Hypothesis test

Taking teachers' teaching leadership as independent variables, learning autonomy, academic self-efficacy and teacher-student relationship as intermediary variables, academic burnout as dependent variables, gender (male = 1, female = 2) and achievement (excellent = 1, medium = 2, poor = 3) as control variables, according to the Bootstrap method recommended by Wen and Ye (2014), the SPSS macro program PROCESS (Model Number = 4; Bootstrap = 2000) as shown in Figure 2 is applied to test the mediating effects of learning autonomy, academic self-efficacy and teacher-student relationship. In advance, the background variables were transformed into virtual variables, and other variables were standardized.

The results of regression analysis show that (Table 2): before the influence of learning autonomy, academic self-efficacy and teacher-student relationship is added, teachers' teaching leadership can negatively predict academic burnout, *R*2 = 0.206, *F*(33, 431) = 38.477, *P* < 0.001, and β = −0.296. Teachers' teaching leadership can positively affect learning autonomy, academic self-efficacy and teacher-student relationship. After adding the influence of learning autonomy, academic self-efficacy and teacher-student relationship, the influence of teachers' teaching leadership on academic burnout is not significant, but work autonomy, professional self-efficacy and professional belonging can all positively affect work passion, which shows that learning autonomy, academic self-efficacy and teacher-student relationship play a completely mediating role in the relationship between teachers' teaching leadership and academic burnout (Wen and Ye, 2014).

**Table 2:** Regression analysis of variable relationship in parallel mediation model.

**Independent variable**	**Learning autonomy**		**Academic self-efficacy**		**Teacher-student relationship**		**Academic burnout**	
	β **value**	**T value**	β **value**	**T value**	β **value**	**T value**	β **value**	**T value**
Gender	-0.150	-2.584*	-0.205	-4.160***	-0.063	-1.580	0.027	0.796
Achievement	-0.209	-5.147***	-0.185	-5.384***	0.030	1.053	0.023	0.665
Teacher's teaching leadership	0.292	7.751***	0.385	12.033***	0.418	6.041***	0.022	0.575
Learning autonomy							-0.307	-7.195***
Academic self-efficacy							-0.468	-9.315***
Teacher-student relationship							-0.115	-2.243***
R2	0.203		0.341		0.377		0.543
Variance ratio	37.906***		76.003***		88.609***		87.035***

^*^p < 0.05, ^***^p < 0.001.

The results of mediation analysis show that (Table 3): Teachers' teaching leadership significantly negatively predicts the academic burnout of senior high school students (β = −0.296, 95%CI[−0.368, −0.225]). In the model test of this equation, *R*2 = 0.206, and *F*(33, 431) = 38.477, P < After adding learning autonomy, academic self-efficacy and teacher-student relationship, the influence of teachers' teaching leadership on academic burnout is not significant, and the indirect effect of learning autonomy on the relationship between teachers' teaching leadership and academic burnout is significant (β = −0.307, 95%CI[−0.391, −0.223], *P* < 0.001). The indirect effect of academic self-efficacy on the relationship between teachers' teaching leadership and academic burnout is also significant (β = −0.468, 95%CI[−0.567, −0.390], *P* < 0.001), and the indirect effect of academic self-efficacy on the relationship between teachers' teaching leadership and academic burnout is also significant (β = −0.115, 95% CI [−0.225, −0.011, *P* < 0.001]. According to the test procedure of mediation effect, the results show that the mediation effects of learning autonomy, academic self-efficacy and teacher-student relationship have all been verified, with the total mediation effect of −0.318 and 95% confidence intervals of [−0.468, −0.286]. Among them, the mediating effect of path 1 “teachers' teaching leadership → learning autonomy → academic burnout” is −0.090, and the 95% confidence interval is [−0.172, −0.050], which does not include 0, indicating that the mediating effect of this path is statistically significant; The mediating effect of Path 2 “Teachers' Teaching Leadership → Academic Self-efficacy → Academic Burnout” is −0.180, and the 95% confidence interval is [−0.306, −0.134], excluding 0, indicating that the mediating effect of this path is statistically significant; The mediating effect of Path 3 “Teachers' Teaching Leadership → Teacher-student Relationship → Academic Burnout” is −0.048, and the 95% confidence interval is [−0.111, −0.002], excluding 0, indicating that the mediating effect of this path is statistically significant.

**Table 3:** General situation of mediating effect test (standard solution).

**Effect category**	**Effect**	**BootSE**	**95 confidence interval**	
			**L**	**U**
Indirect total effect	-0.318	0.047	-0.468	-0.286
Direct effect	0.022	0.038	-0.053	0.096
Total effect	-0.296	0.036	-0.368	-0.225
**Specific indirect effect**				
**Path 1:**				
Leadership → Autonomy → Burnout	-0.090	0.027	-0.172	-0.050
**Path 2:**				
Leadership → Efficiency → Burnout	-0.180	0.036	-0.306	-0.134
**Path 3:**				
Leadership → Teachers and Students → Burnout	-0.048	0.023	-0.111	-0.002

The “Effect” column reports standardized β coefficients for the mediation effects, with significance determined by 95% confidence intervals excluding zero.

## 5 Discussion

### 5.1 The influence of China high school teachers' leadership on students' academic burnout

This study explores the influence of high school teachers' leadership in China on students' academic burnout. The results show that the leadership of high school teachers in China not only has a significant negative impact on students' academic burnout but also has an indirect impact on students' academic burnout through three intermediary variables: learning autonomy, academic efficacy and teacher-student relationship. We calculated that the overall effect of teachers' leadership on students' academic burnout in China was −0.296(95%CI[−0.368, −0.225]). Compared with previous findings where the overall effect of teacher autonomy support on academic burnout was −0.214 (Luo et al., 2014), the effect size observed in our study represents a stronger effect than those reported for other forms of teacher support on academic burnout. This data completely shows that teacher leadership can play a key role in reducing students' academic burnout through providing students' with favorable learning environment and meeting their basic psychological needs. This result is consistent with the previous studies. In school environment, teachers' teaching leadership, as a good social support, helps to reduce the psychological pressure of students and improve their learning motivation (Taylor and Ntoumanis, 2007; Taylor et al., 2008, 2009; Sheldon and Krieger, 2007; Grolnick et al., 2007), to improve self-efficacy (Zhuang et al., 2016), and can also reduce the degree of students' learning burnout (Wentzel et al., 2010; Liao, 2010). In recent years, China has launched a series of policies such as “double reduction” to reduce students' academic burden and their academic burnout. Teachers are not only the implementers of the “double reduction” policy, but also the main participants of “reducing burdens and improving quality” (Song et al., 2023). Therefore, teachers' leadership can promote students' learning (Cowan and Goldhaber, 2016), continuously improve the professional quality of teachers' community members (Harris and Muijs, 2004), and constantly improve the school's educational practice (Wenner and Campbell, 2017), thus effectively reducing students' academic burnout.

### 5.2 The mediating role of learning autonomy, academic efficacy and teacher-student relationship

par Based on the theory of self-determination, this study puts teachers' teaching leadership into one of the influencing factors on students' academic burnout, and further discusses the mechanism of learning autonomy, academic efficacy and teacher-student relationship. The results show that the three factors respectively play an independent intermediary role in the relationship between teachers' teaching leadership and students' academic burden, and hypotheses 2-4 in this study are supported by empirical data. Therefore, the research verified the research framework and hypothesis model, which not only proves the rationality of the analysis framework of this study, but also shows that the influence of external factors on students' academic burden is mainly realized by meeting students' basic psychological needs. In other words, only when external environmental factors able to meet students' psychological needs can academic burnout be effectively reduced.

This study verifies the parallel mediating role of learning autonomy, academic self-efficacy and teacher-student relationship between teachers' teaching leadership and students' academic burnout, thus providing empirical support for the independent perspective study of one-dimensional basic psychological needs. From an independent perspective, the satisfaction of each basic psychological need has independent functional value for students' academic burnout, which is helpful to reduce students' academic burnout. From the perspective of synergy, there is integration or interaction between the three needs, which satisfies the integrated influence on individuals. From the perspective of balance, it expands the finiteness and boundedness of one-dimensional “quality” and holistic “quantity”. This provides empirical support for the idea that the three basic psychological needs put forward by Ryan and Deci (2020) require value of independent, systematic, and balanced functions. In addition, this study also found that among the influences on students' academic burnout, academic efficacy has the greatest predictive power, followed by learning autonomy, and the weakest is the teacher-student relationship.

Based on the self-determination theory, this study systematically integrates five variables: teaching leadership, learning autonomy, academic efficacy, teacher-student relationship and academic burnout. This paper constructs an analytical model to explore how teachers' teaching leadership affects students' academic burnout, and analyzes the relationship and mechanism of these variables through empirical data. The results show that teacher leadership has a significant negative impact on students' academic burnout, and this impact can be further exerted through three intermediary variables: learning autonomy, academic efficacy and teacher-student relationship. This discovery reveals that the generation and persistence of students' academic burnout is the result of the interaction between external environmental factors and internal psychological factors. In addition, the study not only confirms that teacher leadership has a negative predictive effect on students' basic psychological needs-autonomy, competence and relevance-among organizational factors, but also expands the application scope of self-determination theory. The study further verified the independent functional value of autonomy, competence and relevance in alleviating students' academic burnout, thus enriching the understanding of “independence” in the study of one-dimensional basic psychological needs, and confirming the applicability and rationality of self-determination theory in explaining academic burnout.

## 6 Conclusion and prospect

In this study, we found that there is a significant negative correlation between teaching leadership and students' academic burnout. Specifically, the stronger the teacher's teaching leadership, the lower the degree of students' academic burnout. In addition, the role of teaching leadership is not limited to directly reducing academic burnout, but also has a negative impact on students' academic burnout by promoting learning autonomy, enhancing academic efficacy and improving the relationship between teachers and students. These findings emphasize the important role of teacher leadership in shaping a positive learning environment, enhancing students' internal motivation and reducing burnout.

Although this study has made a series of important findings, there are still some limitations. First of all, the findings based on cross-sectional data from Jiangxi Province, China, may have limited generalizability and replicability due to geographic sample constraints and the cross-sectional design. Future research should expand the sample scope and employ longitudinal designs to verify the results. Secondly, while the study confirmed the independent roles of autonomy, competence, and relatedness in linking teaching leadership to reduced student academic burnout, it did not explore their interplay or potential chain mediation effects. Future work should analyze the interactions among these needs and their chain mediating pathways in mitigating academic burnout to better understand the importance of satisfying basic psychological needs.

## Data Availability

The original contributions presented in the study are included in the article/Supplementary material, further inquiries can be directed to the corresponding author.
