# Supplementary material for: The influence of teacher leadership on students academic burnout in China: potential multiple mediating effects of meeting basic psychological needs
Source: Front Psychol. 2025 Aug 27;16:1558159. doi: 10.3389/fpsyg.2025.1558159 (PMC12424051; doi:10.3389/fpsyg.2025.1558159)
Supplement: Supplementary file 1 [file Data_Sheet_1.pdf]

Supplementary Materials

Appendix A

Please read the questions carefully and mark "√" under the appropriate option based on your learning situation. There are no right or wrong answers. Please answer truthfully and do not skip any questions. Thank you.

1. I believe in my ability to achieve good results in my studies1.

Disagree ○1 ○2 ○3 ○4 Agree

2. I believe I am capable of solving the problems I encounter in my studies

Disagree ○1 ○2 ○3 ○4 Agree

3. I am a good learner compared to the rest of the class

Disagree ○1 ○2 ○3 ○4 Agree

4. I believe that I am able to keep up to date with what the teacher is teaching me in the classroom

Disagree ○1 ○2 ○3 ○4 Agree

5. I think I can apply what I have learned.

Disagree ○1 ○2 ○3 ○4 Agree

6. I have a broader knowledge of my field of study than other students in my class

Disagree ○1 ○2 ○3 ○4 Agree

7. I like to choose challenging learning tasks

Disagree ○1 ○2 ○3 ○4 Agree

8. I believe that I can understand well what is written in books and what is taught by teachers

Disagree ○1 ○2 ○3 ○4 Agree

9. I often choose learning tasks that are difficult but can be learned from, even if they require more effort.

Disagree ○1 ○2 ○3 ○4 Agree

10. even if I did very poorly in a particular test, I can calmly analyze the mistakes I made in the test

Disagree ○1 ○2 ○3 ○4 Agree

11. Whether my academic performance is good or bad, I never doubt my ability to learn

Disagree ○1 ○2 ○3 ○4 Agree

12. When studying, I always like to check whether I have mastered what I have learned by asking myself questions.

Disagree ○1 ○2 ○3 ○4 Agree

13. When I think about an issue I am able to make connections between what I have learned and what I have learned before and after

Disagree ○1 ○2 ○3 ○4 Agree

14. I often find myself reading a book without knowing what it means.

Disagree ○1 ○2 ○3 ○4 Agree

15. When reading books I am able to think about what I read in relation to what I already know

Disagree ○1 ○2 ○3 ○4 Agree

16. I find that I am always distracted in class so that I am unable to listen attentively.

Disagree ○1 ○2 ○3 ○4 Agree

17. I often fail to summarize accurately the main meaning of what I read.

Disagree ○1 ○2 ○3 ○4 Agree

18. . I always underline key sections in my books or notebooks to help with studying

Disagree ○1 ○2 ○3 ○4 Agree

19. When I revise for exams, I am able to integrate what I have learned before and after the exams

Disagree ○1 ○2 ○3 ○4 Agree

20. When taking notes in class I always try to write down everything the teacher says, regardless of whether it makes sense or not.

Disagree ○1 ○2 ○3 ○4 Agree

21. When I do my homework, I always try to recall what the teacher has taught me in class in order to do it well

Disagree ○1 ○2 ○3 ○4 Agree

22. Even if the teacher doesn't ask me to, I consciously do the exercises at the end of each chapter in the book to test my knowledge.

Disagree ○1 ○2 ○3 ○4 Agree

23. I was able to devote myself to my studies with great energy.

Disagree ○1 ○2 ○3 ○4 ○5 Agree

24. Recently, I have been feeling very empty and do not know what to do.

Disagree ○1 ○2 ○3 ○4 ○5 Agree

25. I'm so bad at school that I really want to give up.

Disagree ○1 ○2 ○3 ○4 ○5 Agree

26. I am able to reach my goals on a regular basis.

Disagree ○1 ○2 ○3 ○4 ○5 Agree

27. At the end of the day's study, I felt extremely tired.

Disagree ○1 ○2 ○3 ○4 ○5 Agree

28. I don't think it matters whether I learn or not if I don't understand anyway.

Disagree ○1 ○2 ○3 ○4 ○5 Agree

29. When studying, I forget everything around me.

Disagree ○1 ○2 ○3 ○4 ○5 Agree

30. In recent times, I have often felt exhausted.

Disagree ○1 ○2 ○3 ○4 ○5 Agree

31. I do not feel a sense of achievement in my studies.

Disagree ○1 ○2 ○3 ○4 ○5 Agree

32. I don't think studying means anything to me.

Disagree ○1 ○2 ○3 ○4 ○5 Agree

33. I was able to cope well with the examination.

Disagree ○1 ○2 ○3 ○4 ○5 Agree

34. At school, I often feel exhausted.

Disagree ○1 ○2 ○3 ○4 ○5 Agree

35. I studied with a cynical attitude.

Disagree ○1 ○2 ○3 ○4 ○5 Agree

36. I can effectively solve problems that arise in my own learning.

Disagree ○1 ○2 ○3 ○4 ○5 Agree

37. I have always been able to cope easily with academic problems.

Disagree ○1 ○2 ○3 ○4 ○5 Agree

38. It is easy for me to grasp what I have learned.

Disagree ○1 ○2 ○3 ○4 ○5 Agree

39. I care a lot about my teachers

Agree ○1 ○2 ○3 ○4 ○5 Disagree

40. All my teachers liked me.

Agree ○1 ○2 ○3 ○4 ○5 Disagree

41. Teachers' love for students

Agree ○1 ○2 ○3 ○4 ○5 Disagree

42. Teachers often satirize students

Agree ○1 ○2 ○3 ○4 ○5 Disagree

43. Some teachers hit their students

Agree ○1 ○2 ○3 ○4 ○5 Disagree

44. Teachers often argue with students

Agree ○1 ○2 ○3 ○4 ○5 Disagree

45. Teachers complaining to parents behind their backs

Agree ○1 ○2 ○3 ○4 ○5 Disagree

46. Pupils are afraid of and resentful of their teachers.

Agree ○1 ○2 ○3 ○4 ○5 Disagree

47. Teachers are always found to be working too hard every day

Agree ○1 ○2 ○3 ○4 ○5 Disagree

48. Fear of mistakes and criticism by teachers

Agree ○1 ○2 ○3 ○4 ○5 Disagree

49. I enjoy going to all the teachers' classes

Agree ○1 ○2 ○3 ○4 ○5 Disagree

50. I particularly admire teachers

Agree	○1	○2	○3	○4	○5	Disagree

51. Teachers are very fair to students

Agree	○1	○2	○3	○4	○5	Disagree

52. Teachers often lose their temper for no apparent reason.

Agree	○1	○2	○3	○4	○5	Disagree

53. Frequent nicknames for teachers

Agree	○1	○2	○3	○4	○5	Disagree

54. Teachers only care about students who are good learners

Agree	○1	○2	○3	○4	○5	Disagree

55. Teachers talking about students behind their backs

Agree	○1	○2	○3	○4	○5	Disagree

56. Teachers often try to withdraw poor students from school

Agree	○1	○2	○3	○4	○5	Disagree

57. Great admiration for teachers

Agree	○1	○2	○3	○4	○5	Disagree

58. Dissatisfaction with their relationship with teachers

Agree	○1	○2	○3	○4	○5	Disagree

59. Always want to be praised by teachers

Agree	○1	○2	○3	○4	○5	Disagree

60. Reluctance to engage with teachers

Agree ○1 ○2 ○3 ○4 ○5 Disagree

61. I believe I have better control over my studies.

Agree ○1 ○2 ○3 ○4 ○5 Disagree

62. With the help of others, I am able to rationalize my learning needs.

Agree ○1 ○2 ○3 ○4 ○5 Disagree

63. I was able to understand the difference between being forced to learn by a teacher and learning on my own initiative.

Agree ○1 ○2 ○3 ○4 ○5 Disagree

64. I am able to use my peers as helpers and encouragers of my own learning.

Agree ○1 ○2 ○3 ○4 ○5 Disagree

65. I am able to translate my learning needs into learning objectives and take action.

Agree ○1 ○2 ○3 ○4 ○5 Disagree

66. I am able to recognize effective learning strategies and have the ability to apply them.

Agree ○1 ○2 ○3 ○4 ○5 Disagree

67. I am able to identify learning resources that will help me accomplish my learning goals.

Agree ○1 ○2 ○3 ○4 ○5 Disagree

68. I am able to gather evidence that I am making progress towards the goals I have set for myself

Agree ○1 ○2 ○3 ○4 ○5 Disagree

69. I am able to learn what I want on my own, without external demands, school rules or pressure from others.

Agree ○1 ○2 ○3 ○4 ○5 Disagree

70. I am able to reflect on and improve my learning behaviors and learning methods

Agree ○1 ○2 ○3 ○4 ○5 Disagree

71. I have the flexibility to vary my reading speed according to the purpose of the reading.

Agree ○1 ○2 ○3 ○4 ○5 Disagree

72. When listening to lectures, I am able to take notes in a structured manner, focusing on the main points, rather than recording everything from beginning to end.

Agree ○1 ○2 ○3 ○4 ○5 Disagree

73. My teachers are able to determine the students' need for learning at the beginning of the course.

Agree ○1 ○2 ○3 ○4 ○5 Disagree

74. My teacher is able to communicate educational objectives and learning goals through a good course syllabus.

Agree ○1 ○2 ○3 ○4 ○5 Disagree

75. My teacher discusses with students about course objectives, individual expectations and needs, etc.

Agree ○1 ○2 ○3 ○4 ○5 Disagree

76. My teacher's instructional goals take into account the maturity of the students.

Agree ○1 ○2 ○3 ○4 ○5 Disagree

77. My teachers are clear about their educational philosophy and apply it in their teaching.

Agree ○1 ○2 ○3 ○4 ○5 Disagree

78. My teachers consider learning to be a valuable activity in itself.

Agree ○1 ○2 ○3 ○4 ○5 Disagree

79. My teachers are able to see themselves as aids in the learning process of their students.

Agree ○1 ○2 ○3 ○4 ○5 Disagree

80. My teacher promotes students' identification of their self-worth in an encouraging way.

Agree ○1 ○2 ○3 ○4 ○5 Disagree

81. My teachers care about the learning process of their students.

Agree ○1 ○2 ○3 ○4 ○5 Disagree

82. My teachers hold students accountable for their own learning.

Agree ○1 ○2 ○3 ○4 ○5 Disagree

83. During classes, the teacher's concept of organization is very clear.

Agree ○1 ○2 ○3 ○4 ○5 Disagree

84. My teacher encourages or praises the effort students put into their studies.

Agree ○1 ○2 ○3 ○4 ○5 Disagree

85. My teacher will keep repeating the learning objectives, the value of the program, etc. to show students the importance of learning.

Agree ○1 ○2 ○3 ○4 ○5 Disagree

86. My teacher is able to encourage students to take an active part in the learning process.

Agree ○1 ○2 ○3 ○4 ○5 Disagree

87. My teacher is able to listen to students' problems with an open and receptive attitude.

Agree ○1 ○2 ○3 ○4 ○5 Disagree

88. When students are faced with a problem that cannot be solved, my teachers are able to guide students to find alternatives to solve the problem.

Agree ○1 ○2 ○3 ○4 ○5 Disagree

89.My teacher will adjust the lessons to meet the needs of the students.

Agree ○1 ○2 ○3 ○4 ○5 Disagree

90. My teacher is able to maintain good teacher-student communication with students.

Agree	○1	○2	○3	○4	○5	Disagree

91. My teachers create a congenial teaching atmosphere.

Agree	○1	○2	○3	○4	○5	Disagree

92. My teacher is able to guide students through peer learning or other means.

Agree	○1	○2	○3	○4	○5	Disagree

93. My teacher is able to guide students in the learning process.

Agree	○1	○2	○3	○4	○5	Disagree

94. My teachers will integrate student experiences into their teaching.

Agree	○1	○2	○3	○4	○5	Disagree

95. My teachers will enhance mutual trust and respect between teachers, students and peers.

Agree	○1	○2	○3	○4	○5	Disagree

96. My teachers encourage students to think independently.

Agree	○1	○2	○3	○4	○5	Disagree

97. My teachers would encourage students to take risks and be courageous.

Agree	○1	○2	○3	○4	○5	Disagree
